# Preoperative lymphocyte-to-monocyte ratio as a strong predictor of survival and recurrence for gastric cancer after radical-intent surgery

**DOI:** 10.18632/oncotarget.17058

**Published:** 2017-04-12

**Authors:** Jun-Peng Lin, Jian-Xian Lin, Long-Long Cao, Chao-Hui Zheng, Ping Li, Jian-Wei Xie, Jia-Bin Wang, Jun Lu, Qi-Yue Chen, Mi Lin, Ru-Hong Tu, Chang-Ming Huang

**Affiliations:** ^1^ Department of Gastric Surgery, Fujian Medical University Union Hospital, Fuzhou 350001, Fujian Province, China

**Keywords:** gastric cancer, preoperative lymphocyte-to-monocyte ratio, nomogram, overall survival, recurrence

## Abstract

**Objectives:**

To evaluate the predictive value of the preoperative lymphocyte-to-monocyte ratio (LMR) for the prognosis of patients with gastric cancer (GC) after radical-intent surgery.

**Methods:**

We retrospectively analyzed 1,810 patients who underwent radical-intent gastrectomy for primary GC from December 2008 to December 2013. X-tile software was used to identify the optimal value for blood LMR. Nomograms were developed to predict overall survival (OS) and recurrence-free survival (RFS) after surgery.

**Results:**

LMR was significantly lower in patients with GC than in matched normal volunteers (P<0.001). As shown by forest plots, the long-term outcomes were poorer in the low LMR group than in the high LMR group when considering subgroups separated by clinical characteristics. Cox regression analysis showed that LMR was an independent prognostic factor for OS (P<0.001) and RFS (P=0.001). Nomograms, combining LMR with age, T stage, and N stage, showed better discriminative abilities than the AJCC staging system did in predicting 5-year survival and recurrence from the time of surgery. The recurrence rate was 30.4% (550/1810) and was significantly higher in the low LMR group than in the high LMR group (P<0.05). The LMR was also closely correlated with liver and lymph node metastases (both P<0.05).

**Conclusion:**

As an independent prognostic factor for GC, preoperative LMR can improve the predictability of individual survival and recurrence. Furthermore, because liver and lymph node metastases were more commonly observed in patients with low blood LMR before surgery, these patients should be closely followed after the operation.

## INTRODUCTION

Gastric cancer (GC) is one of the most common cancer and the second leading cause of cancer-related death worldwide [1]. In recent decades, significant progress has been made in surgical techniques and adjuvant therapy; however, the prognosis of patients with GC remains poor [2]. Proposed by the American Joint Committee on Cancer (AJCC) [3], the TNM scoring system uses the pathological depth of invasion and the number of metastatic lymph nodes as important prognostic factors for patients with GC. However, there remain some differences in prognosis among patients with the same tumor stage. Thus, how to develop an individual treatment plan according to the tumor characteristics and patient factors remains a main concern in the treatment of GC.

Recently, the systemic inflammatory response was shown to be associated with worse prognosis in multiple tumors [4]. One previous study showed that tumor-associated macrophages (TAMs) derived from peripheral blood mononuclear cells play a key role in promoting tumor progression and metastasis in the tumor microenvironment [5]. Recently, it was reported that the preoperative blood lymphocyte-to-monocyte ratio (LMR) seems to be used as a prognostic indicator for solid tumors [6]. However, evidence for the predictive value of the blood LMR in GC remains poor and sufficiently large sample sizes are lacking [7]. The aims of this study were to investigate the predictive value of the preoperative blood LMR for the long-term prognosis of patients with GC after radical-intent surgery and to construct a novel predictive model.

## RESULTS

### Blood LMR was lower in patients with GC

In total, 501 NVs were enrolled, with the NV group presenting similar age and gender proportion as the GC group (both P>0.05, Figure 2A). Lymphocyte count and LMR were significantly lower in patients with GC than in NVs (P<0.001, Figure 2B and 2D), whereas monocyte count was significantly higher in GC patients than in NVs (P<0.001, Figure 2C).

**Figure 1:**
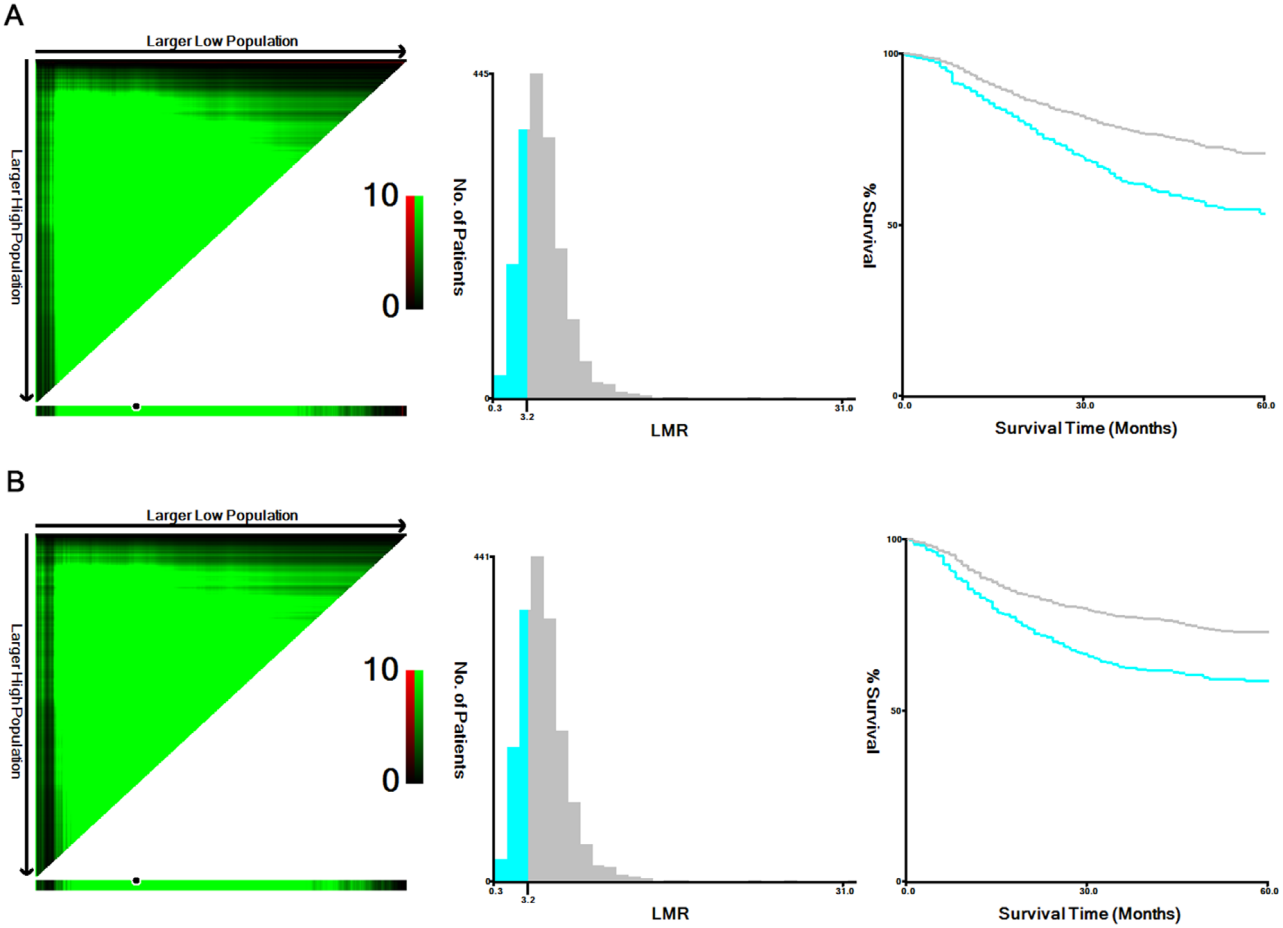
X-tile analyses of 5-year OS **(A)** and RFS **(B)** performed using patient data to determine the optimal cut-off value for blood LMR. In the left panels, the X-axis represents all potential cut-off values from low to high (left to right) that define a low subset, whereas the Y-axis represents the cut-off values from high to low (top to bottom) that define a high subset. Red coloration of a cut-off value indicates an inverse correlation with time to recurrence, and green coloration represents direct associations. The optimal cut-off values highlighted by the black circles in the left panels are shown in the histograms of the entire cohort (middle panels). Kaplan-Meier plots are displayed in the right panels, where blue represents the low subgroup and gray represents the high subgroup. The optimal cut-off value for blood LMR is 3.15 for both OS and RFS.

**Figure 2:**
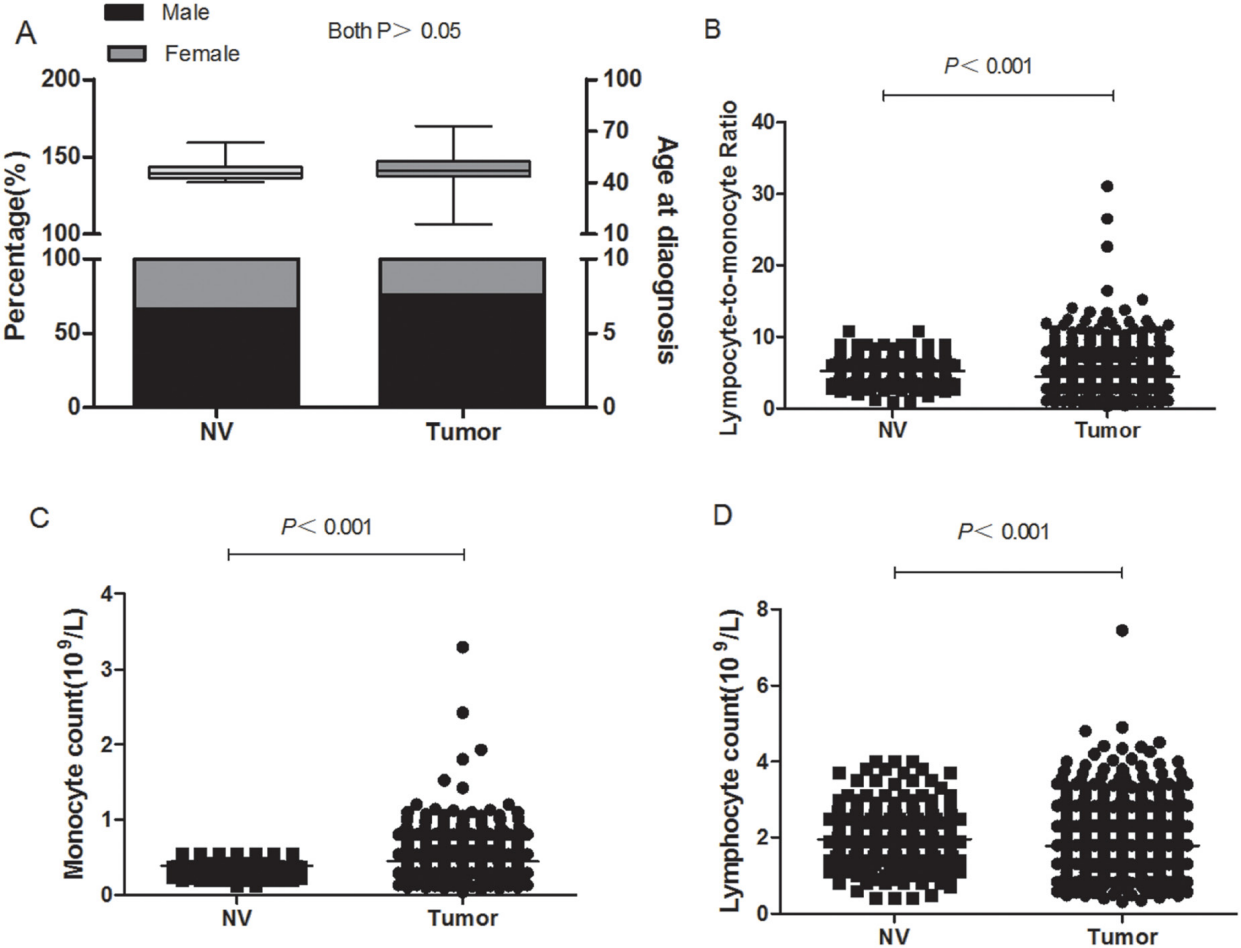
Blood cell counts from normal volunteers and patients with GC **(A)** There was no significant difference in age and gender between NVs and patients with GC (both P>0.05). **(B)** The blood LMR in patients with GC was significantly lower than that in NVs (4.51±0.05 vs. 5.26±0.06, P<0.05). **(C)** The monocyte counts in patients with GC were significantly higher than those in NVs (0.44±0.46 vs. 0.38±0.01, P<0.05). **(D)** The lymphocyte counts of GC patients were significantly lower than those of the NVs (1.78±0.02vs 1.96±0.02, P<0.05).

### Clinicopathologic characteristics of the patients

There were 1,358 cases in the training set and 452 cases in the validation set, and the clinical pathological characteristics were no significant differences between the two groups (P>0.05). Among all of the patients, the 30-day mortality rate in both the patients with low LMR and those with high LMR was 0.2% (P>0.05). For the training set, the relationships between each of the clinicopathologic characteristics and LMR are given in Table 1. Patients with low LMR were more frequently men older than 65 years than patients with high LMR (both P<0.001). We found that patients with low LMR were more likely to have a large tumor size than were high LMR patients and more likely to have higher T stages and N stages (P<0.05 for all). And patients with vascular invasion and perineural invasion had lower LMR (both P<0.05). Adjuvant chemotherapy, complications and total gastrectomy were significantly more common in the low LMR group (P<0.05 for all). However, the other clinical pathological datas were not associated with the LMR (all P>0.05).

**Table 1:** Patient baseline clinicopathologic characteristics

Clinicopathological feature	Training Set				Validation Set Total (n=452)	P
	Low LMR (n=365)	High LMR (n=993)	P	Total (n=1358)		
Age, n (%)			<0.001			0.131
<65	167 (45.8)	653 (65.8)		820 (60.4)	291 (64.4)	
≥65	198 (54.2)	340 (34.2)		538 (39.6)	161 (35.6)	
Gender, n (%)			<0.001			0.887
Male	302 (82.7)	730 (73.5)		1032 (76.0)	342 (75.7)	
Female	63 (17.3)	263 (26.5)		326 (24.0)	110 (24.3)	
Site, n (%)			<0.001			0.279
Upper	134 (36.7)	312 (31.4)		446 (32.8)	161 (35.6)	
No upper	231 (63.3)	681 (68.6)		912 (67.2)	291 (64.4)	
T stage, n (%)			<0.001			0.372
T1	52 (15.3)	287 (28.9)		339 (25.0)	108 (23.9)	
T2	30 (20.8)	114 (11.5)		144 (10.6)	55 (12.2)	
T3	117 (29.4)	281 (28.3)		398 (29.3)	146 (32.3)	
T4	166 (34.8)	311 (31.3)		477 (35.1)	143 (31.6)	
N stage, n (%)			<0.001			0.355
N0	86 (23.6)	427 (43.0)		513 (37.8)	174 (38.5)	
N1	48 (13.2)	155 (15.6)		203 (14.9)	79 (17.5)	
N2	74 (20.3)	155 (15.6)		229 (16.9)	63 (13.9)	
N3	157 (43.0)	256 (25.8)		413 (30.4)	136 (30.1)	
Vascular invasion, n (%)			<0.001			0.908
Negative	244 (66.8)	810 (76.9)		1054 (77.6)	352 (7.9)	
Positive	121 (33.2)	183 (18.4)		304 (22.4)	100 (22.1)	
Perineural invasion, n (%)			0.027			0.888
Negative	297 (81.4)	856 (86.2)		1153 (84.9)	385 (85.2)	
Positive	68 (18.6)	137 (13.8)		205 (15.1)	67 (14.8)	
Tumor size (cm), n (%)			0.004			0.129
≤5.0	185 (50.7)	589 (59.3)		774 (57.0)	276 (61.1)	
>5.0	180 (49.3)	404 (40.7)		584 (43.0)	176 (38.9)	
Tumor grade, n (%)			0.896			0.450
G1	20 (5.5)	56 (5.6)		76 (5.6)	31 (6.9)	
G2	211 (57.8)	552 (55.6)		763 (56.2)	260 (57.5)	
G3	132 (36.2)	378 (38.1)		510 (37.6)	160 (35.4)	
G4	2 (0.5)	7 (0.7)		9 (0.7)	1 (0.2)	
Histological type, n (%)			0.731			0.749
Differentiated	74 (20.3)	193 (19.4)		267 (19.7)	92 (20.4)	
Undifferentiated	291 (79.7)	800 (80.6)		1091 (80.3)	360 (79.6)	
Margin status, n (%)			0.750			0.296
Negative	361 (98.9)	984 (99.1)		1345 (99.0)	450 (99.6)	
Positive	4 (1.1)	9 (69.2)		13 (1.0)	2 (0.4)	
Type of gastrectomy, n (%)			<0.001			0.724
Subtotal	156 (42.7)	532 (53.6)		688 (50.7)	233 (51.5)	
Total	209 (57.3)	461 (46.4)		670 (49.3)	219 (48.5)	
LNs resected, median±SD	32.8±12.7	33.7±13.2	0.109	33.5±13.1	33.4±12.9	0.916
Extent of lymphadenectomy			0.142			0.824
D1	49 (13.4)	105 (10.6)		154 (11.3)	53 (11.7)	
D2	316 (86.6)	888 (89.4)		1204 (88.7)	399 (88.3)	
Adjuvant chemotherapy, n (%)			<0.001			0.938
Yes	261 (71.5)	574 (57.8)		835 (61.5)	277 (61.3)	
No	104 (28.5)	419 (42.2)		523 (38.5)	175 (38.7)	
Complications, n (%)			0.023			0.539
Yes	74 (20.3)	150 (15.1)		224 (16.5)	69 (15.3)	
No	291 (79.7)	843 (84.9)		1134 (83.5)	383 (84.7)	
30-day mortality, n (%)	4 (1.1)	3 (0.3)	0.167	7 (0.5)	0 (0.0)	0.126

### Low blood LMR was associated with poor prognosis

**Figure 3:**
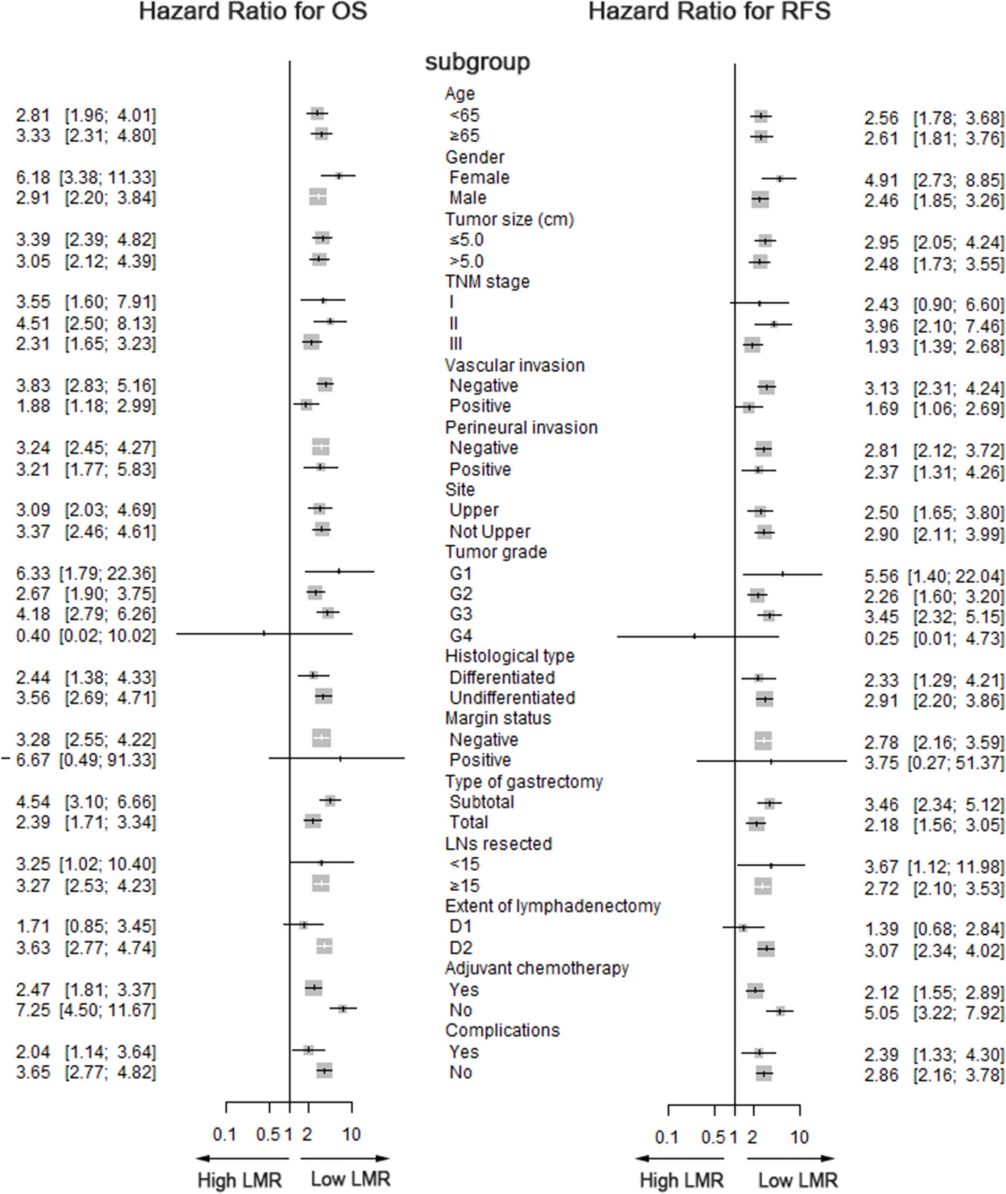
Forest plot showing OS and RFS according to subgroup effects

The median follow-up was 41 months (range 1-92 months). Patients with high LMR had higher 3-year and 5-year cancer-specific survival rates relative to the low LMR group (77.6% vs. 62.1% and 68.5% vs. 50.5%; both P<0.05, Supplementary Figure 2). Low LMR was also associated with reduced OS and RFS (P<0.05 for all; Supplementary Figure 2). For patients classified as stage I, stage II or stage III, low LMR was significantly associated with worse OS, CSS and RFS (P<0.05 for all; Supplementary Figure 2). Figure 3 shows the OS and RFS results, with OS and RFS analyzed according to age, gender, tumor site, tumor size, grade, TNM stage, vascular invasion, perineural invasion, histological type, margin status, type of gastrectomy, LNs resected, extent of lymphadenectomy, adjuvant chemotherapy, complications and LMR. The hazard ratio and 95% confidence interval (CI) for OS and RFS were compared among the subgroups. The long-term survival rates, including OS and RFS, were poorer in patients with low LMR than in those with high LMR for all subgroups.

### Prognostic value of LMR

A univariate survival analysis and a multivariate survival analysis were used to analyze the factors that influenced OS and RFS. The univariate analyses showed that age, tumor site, T stage, N stage, vascular invasion, perineural invasion, tumor size, tumor grade, type of gastrectomy, comorbidities and LMR were associated with OS and RFS (P<0.05 for all; Table 2). The multivariate analysis of these variables (significant in the univariate analyses) showed that only LMR, age, T stage and N stage were independently correlated with OS and RFS (P<0.05 for all; Table 3).

**Table 2:** Univariate analysis of clinicopathologic variables in relation to OS and RFS in patients with GC undergoing radical-intent resection

Clinicopathological feature	OS		RFS	
	HR (95% CI)	P	HR (95% CI)	P
Age		<0.001		<0.001
<65	1 (Referent)		1 (Referent)	
≥65	1.689 (1.402-2.035)		1.596 (1.312-1.942)	
Gender		0.702		0.812
Male	1 (Referent)		1 (Referent)	
Female	0.958 (0.770-1.193)		0.972 (0.772-1.224)	
Site		<0.001		<0.001
Upper	1 (Referent)		1 (Referent)	
No upper	0.703 (0.581-0.851)		0.670 (0.549-0.819)	
T stage		<0.001		<0.001
T1	1 (Referent)		1 (Referent)	
T2	2.357 (1.321-4.203)		3.193 (1.655-6.163)	
T3	6.313 (4.091-9.741)		8.045 (4.777-13.549)	
T4	11.744 (7.723-17.860)		15.777 (9.505-26.190)	
N stage		<0.001		<0.001
N0	1 (Referent)		1 (Referent)	
N1	1.834 (1.243-2.707)		1.836 (1.181-2.855)	
N2	3.312 (2.381-4.606)		3.811 (2.650-5.480)	
N3	8.373 (6.355-11.031)		10.027 (7.366-13.651)	
Vascular invasion		<0.001		<0.001
Negative	1 (Referent)		1 (Referent)	
Positive	1.810 (1.476-2.218)		1.856 (1.501-2.295)	
Perineural invasion		<0.001		<0.001
Negative	1 (Referent)		1 (Referent)	
Positive	1.620 (1.281-2.050)		1.544 (1.213-1.991)	
Tumor size (cm)		<0.001		<0.001
≤5.0	1 (Referent)		1 (Referent)	
>5.0	1.845 (1.530-2.224)		1.973 (1.619-2.405)	
Tumor grade		0.372		0.332
G1	1 (Referent)		1 (Referent)	
G2	0.982 (0.646-1.495)		1.052 (0.672-1.645)	
G3	1.108 (0.723-1.698)		1.138 (0.721-1.795)	
G4	1.836 (0.700-4.811)		2.271 (0.856-6.024)	
Histological type		0.216		0.138
Differentiated	1 (Referent)		1 (Referent)	
Undifferentiated	1.167 (0.914-1.490)		1.219 (0.939-1.582)	
Margin status		0.562		0.828
Negative	1 (Referent)		1 (Referent)	
Positive	1.298 (0.537-3.134)		1.115 (0.417-2.987)	
Type of gastrectomy		<0.001		<0.001
Subtotal	1 (Referent)		1 (Referent)	
Total	1.802 (1.490-2.180)		1.903 (1.555-2.329)	
LNs resected		0.111		0.069
<15	1 (Referent)		1 (Referent)	
≥15	0.711 (0.467-1.082)		0.665 (0.429-1.032)	
Extent of lymphadenectomy		0.637		0.608
D1	1 (Referent)		1 (Referent)	
D2	0.933 (0.689-1.246)		0.924 (0.683-1.249)	
Adjuvant chemotherapy		0.058		0.734
Yes	1 (Referent)		1 (Referent)	
No	0.833 (0.689-1.006)		0.966 (0.789-1.181)	
Complications		0.019		0.048
No	1 (Referent)		1 (Referent)	
Yes	1.329 (1.048-1.686)		1.288 (1.002-1.656)	
LMR		<0.001		<0.001
Low (≤3.15)	1 (Referent)		1 (Referent)	
High (>3.15)	0.334 (0.276-0.403)		0.361 (0.296-0.441)	

**Table 3:** Multivariate analysis of clinicopathologic variables in relation to OS and RFS in patients with GC undergoing radical-intent resection

Clinicopathological feature	OS		RFS	
	HR (95% CI)	P	HR (95% CI)	P
Age		<0.001		<0.001
<65	1 (Referent)		1 (Referent)	
≥65	1.606 (1.328-1.942)		1.452 (1.189-1.773)	
T stage		<0.001		<0.001
T1	1 (Referent)		1 (Referent)	
T2	1.934 (1.069-3.499)		2.600 (1.326-5.098)	
T3	3.080 (1.910-4.965)		3.606 (2.043-6.364)	
T4	4.994 (3.109-8.023)		5.887 (3.347-10.356)	
N stage		<0.001		<0.001
N0	1 (Referent)		1 (Referent)	
N1	1.174 (0.784-1.757)		1.131 (0.717-1.783)	
N2	1.629 (1.140-2.326)		1.827 (1.236-2.701)	
N3	3.557 (2.590-4.885)		4.164 (2.929-5.920)	
LMR		<0.001		<0.001
Low (≤3.15)	1 (Referent)		1 (Referent)	
High (>3.15)	0.473 (0.390-0.572)		0.515 (0.421-0.631)	

**Figure 4:**
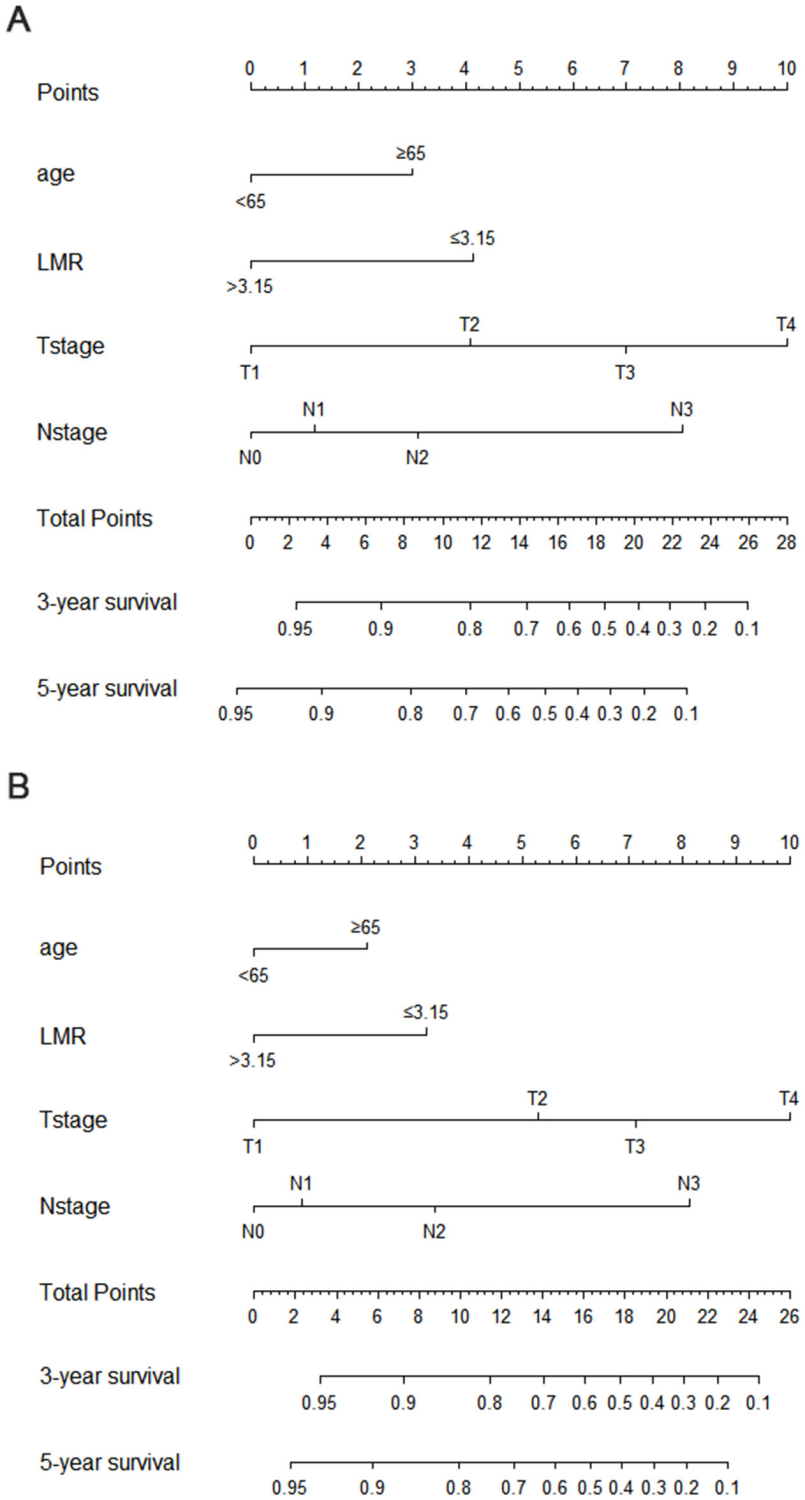
Nomogram to estimate the probability of OS **(A)** and RFS **(B)** at 3 and 5 years.

Prognostic nomograms and their calibration curves were established with R software (Figure 4 and Supplementary Figure 3, respectively). The C-index of the nomograms for OS and RFS, including age, LMR, T stage and N stage, were 0.790 and 0.795, respectively. The C-index of the AJCC staging system was also calculated for OS and RFS and was 0.719 and 0.727, respectively. Similarly, the nomogram for the validation set had higher C-index than the AJCC staging system (C-index for OS, 0.751 vs. 0.721; C-index for RFS, 0.779 vs. 0.697). Together, these data showed that our nomograms had a superior ability to predict 3- and 5-year OS and RFS for patients with GC compared with the AJCC staging system, indicating the value of this prognostic prediction system for patients with GC after radical-intent operation.

### Blood LMR was significantly correlated with recurrence

The recurrence rate was 30.5% (550/1,803) (exclusion of 30-day mortality). Patients with low LMR had higher recurrence rate relative to the high LMR group (44.2% vs. 22.8%, P<0.05).

Details regarding the recurrence site following surgery are listed in Table 4. A low blood LMR was significantly associated with both liver metastasis and lymph node metastasis compared with a high LMR (liver: 10.6% vs. 6.5%; lymph node: 8.9% vs. 5.9%; both P<0.05). However, the remaining recurrence sites were not related to the LMR (P>0.05 for all).

**Table 4:** Site of recurrence after surgery

Clinicopathological feature	Low LMR (%) n=473	High LMR (%) n=1330	P
Liver	10.6 (50/473)	6.5 (87/1330)	0.005
Peritoneum	8.0 (38/473)	5.8 (77/1330)	0.086
Lymph node	8.9 (42/473)	5.9 (78/1330)	0.024
Lung	3.2 (15/473)	1.9 (25/1330)	0.101
Bone	2.1 (10/473)	1.1 (15/1330)	0.115
Pelvic cavity	3.2 (15/473)	2.3 (30/1330)	0.273
Anastomosis	4.0 (19/473)	2.9 (39/1330)	0.251
Adrenal gland	0.6 (3/473)	0.4 (5/1330)	0.747
Pancreas	0.8 (4/473)	0.5 (6/1330)	0.527
Remnant stomach	1.1 (5/473)	0.3 (4/1330)	0.104
Spleen	0.4 (2/473)	0.2 (2/1330)	0.612
Ovary	0.6 (3/473)	0.5 (6/1330)	0.923
Brain	0.6 (3/473)	0.4 (5/1330)	0.753
Colon	1.1 (5/473)	0.8 (10/1330)	0.538
Esophagus	0.6 (3/473)	0.5 (6/1330)	0.923
Other	1.7 (8/473)	1.0 (13/1330)	0.219

## DISCUSSION

In recent years, increasing numbers of studies have confirmed that the systemic inflammatory response is closely related to tumor progression and metastasis [11, 12]. As one of the indexes reflecting inflammation, an increased blood LMR has been reported in some studies to be closely related to a better prognosis in patients with hematological diseases and solid tumors [13–16]. However, little research has been conducted on the relationship between LMR and the prognosis of GC, and in the few studies available, the sample sizes are small, and the results are inconsistent [17, 18]. The present study represents the single largest consecutive GC cohort used to evaluate the relationship between preoperative LMR and the prognosis of patients with GC. In previous studies, ROC curve analysis, the R package MaxStat, survival tree R software and other methods were used to select the optimal cut-off values for LMR [7, 18, 19]. However, in the present study, we identified optimal cutoffs of LMR for OS and RFS (3.15) using the minimum P value from log-rank χ^2^ statistics with the X-tile program in a cohort including 1,810 GC patients. In fact, the cut-off values of LMR for tumor patients varies among studies. Some studies reported that LMR was associated with pT stage, pN stage, tumor diameter and age [20, 21]. The optimal cutpoint of LMR in our study is different from other studies, which maybe because the aforementioned clinicopathological features in our study are inconsistent with others. Chan et al found that the cutpoints of LMR isn’t similar in different stages [19]. However, our study is mainly aimed at the total GC patients, we did not further analyze the cut-off value of LMR in each stage. X-tile plots present a new tool for the assessment of biological relationships between a biomarker and outcome; and the discovery of population cut-points based on marker expression. A population is divided into different divisions based on every possible cut-off point. All possible divisions of the cut-off point are statistically assessed. Then, X-tile plots calculate χ^2^ values for every possible division of the population. The optimal cut-off value for survival was calculated by selecting the minimum P value with the maximum χ^2^ value [9]. Our results demonstrated that our approach yields greater discriminatory ability and more predictive accuracy than do the approaches in previous studies. Furthermore, we classified patients with a blood LMR lower than the cut-off value as having low LMR, whereas the other subjects were defined as having high LMR.

The effects of LMR on tumor prognosis have been confirmed, with studies showing that preoperative low blood LMR is correlated with poor prognosis in gastrointestinal neoplasia [22]. LMR is composed of lymphocytes and monocytes. Lymphocytes are associated with tumor immunity, while monocytes can promote tumor progression. Some studies have confirmed that LMR has an impact on the prognosis of stage I tumors [19, 23], which is consistent with the results of this study. In GC, Hsu et al. reported that the preoperative LMR independently predicted survival in patients with resectable GC [18]. However, Deng’s results showed that the blood LMR as a preoperative marker was not an independent prognostic indicator for long-term outcome in patients with GC [17]. Thus, the relationship between LMR and prognosis of patients with GC remains unclear. The current study is the first to compare the levels of blood LMR between patients with GC and NVs. Blood LMR was lower in patients with GC than in NVs, whereas the monocyte counts were significantly higher in patients with GC than in NVs. By examining the relationships between LMR and each of several clinicopathologic features, we found that low LMR was correlated with some variables which previously was confirmed to be negative prognostic factors. These variables include higher T stage, higher N stage, perineural invasion, vascular invasion and large tumor size. Therefore, we used a forest plot in a stratified analysis based on clinicopathologic features and found that the prognostic value of LMR was consistent when considering subgroups. Multivariate analysis further revealed that low LMR was also associated with poor prognosis of patients with GC. But the number of lymph node retrieval, extent of lymphadenectomy and adjuvant chemotherapy did not significantly affect overall survival or recurrence. In our study, the number of patients with less than 15 lymph node removed was significantly less than those with greater than or equal 15 lymph node removed (57 vs 1351), which may cause the number of lymph node retrieval did not significantly affect overall survival or recurrence. Patients undergoing D1 lymph node dissection are mostly in the earlier stage than those with D2 in our study. So the extent of lymphadenectomy did not influence OS or RFS, which is consistent with the previous study [24]. In China, patients with adjuvant chemotherapy were more likely to have higher stage than those with non-adjuvant chemotherapy and we didn’t match the stage in recurrence and survival analyses. That would be the reason why adjuvant chemotherapy did not associate with OS or RFS. In clinical practice, nomograms have been proposed as an important tool for the individual prediction of prognosis in patients with cancer [25, 26]. In this study, we established prognostic nomograms for OS and RFS by combining blood LMR, age, T stage, and N stage, and the C-index for OS and RFS was 0.790 and 0.795, respectively, representing more-optimal predictive ability than the AJCC staging system. In addition, we conducted an internal validation test, which further confirmed the predictive efficiency of the nomograms. Therefore, as a novel prognostic system, our nomograms may provide simple, more accurate prognostic prediction. The mechanism for the effect of LMR on tumor prognosis remains unclear. As basic components of the adaptive and innate immune system, lymphocytes play an important role in immunosurveillance and immunoediting [27]. Hoffmann et al. showed that a low quantity of lymphocytes may indicate an insufficient immunologic reaction to the tumor [28], and previous studies have confirmed that lymphocytopenia may lead to reduce survival of patients with cancer [29, 30]. In contrast, The role of monocytes in tumor progression remains controversial [11]. In breast cancer, Evani et al. found that monocytes can enhence adhesion of tumor cells to the endothelium, which may promote cancer metastasis [31]. Moreover, monocytes can differentiate into macrophages which can contribute to tumor metastasis by producing SPARC/osteonectin [31–32]. So as a surrogate marker for high tumor burden, the elevated macrophages can be reflected by the increased monocytes [33]. Recent studies have demonstrated that tumor-associated macrophages (TAMs) play a key role in promoting tumor development, metastasis, angiogenesis, and tumor immunity [5].

In patients with malignant tumors, tumor recurrence and distant metastasis are the main causes of death. Zhou et al. found that preoperative low LMR can improve the postoperative recurrence rate of GC in 426 patients with stage II/III disease [7]. In our study, the 5-year recurrence rate for patients with GC after radical-intent surgery was 37%, and patients with low LMR had higher recurrence rate relative to the high LMR group. Liver and lymph node metastases were the most common types of recurrence, followed by peritoneal metastasis, whereas the spleen, adrenal gland, brain, and other locations were relatively rare sites of recurrence. Therefore, in the course of follow-up, clinicians should pay more attention to the predictive value of preoperative blood LMR for tumor recurrence to identify potential liver or lymph node metastasis as early as possible. Our study showed that low preoperative LMR are associated with more advanced stage, higher recurrence rates and worse prognosis. Detailed follow-up should be performed with such patients, especially to assess whether there is metastasis of the liver and lymph nodes. Such follow-up will allow clinicians to identify recurrence as soon as possible and provide patients with further treatment such as adjuvant chemotherapy to improve the prognosis. Although this was a retrospective case-control study performed within a single institution, it is the first study to comprehensively and systematically confirm that preoperative serum LMR, a simple, easily measurable, and inexpensive inflammatory biomarker, can successfully predict the long-term survival of patients with GC after radical-intent surgery. Thus, our study can serve as the basis for subsequent prospective clinical studies.

## MATERIALS AND METHODS

This study performed a retrospective analysis of a database of 1,810 primary GC patients treated with radical-intent surgery in the Department of Gastric Surgery of Fujian Medical University Union Hospital, Fuzhou, China between December 2008 and December 2013. The inclusion criteria were as follows: (1) a histologically confirmed adenocarcinoma of the stomach; (2) no evidence of tumors invading the adjacent organs (pancreas, spleen, liver, and transverse colon), paraaortic lymph node enlargement or distant metastasis demonstrated by abdominal computed tomography and/or abdominal ultrasound and posteroanterior chest radiographs; and (3) a D1 +α/D1 +β/D2 lymphadenectomy with curative R0 according to the pathological diagnosis after the operation. The exclusion criteria were as follows: (1) no routine blood examination before surgery, (2) metastatic disease, (3) neoadjuvant chemotherapy, (4) malignant disease of other organs, (5) presence of coexisting hematological malignancies or disorders or autoimmune disorders, and (6) incomplete/inaccurate medical records (Supplementary Figure 1). The staging was performed according to the seventh corresponding edition of the AJCC Staging Manual [3]. The type of surgical resection (i.e., distal subtotal gastrectomy, proximal subtotal gastrectomy, or total gastrectomy) and the extent of lymph node dissection were selected according to the Japanese Gastric Cancer Treatment Guidelines [8]. Adjuvant chemotherapy with 6 cycles of capecitabine plus oxaliplatin (XELOX) or TS-1 plus oxaliplatin (SOX) was recommended to all patients with advanced GC, and most of the patients were treated with the regimen of adjuvant chemotherapy. Most of the patients with recurrence were treated with paclitaxel plus 5-fluorouracil as second-line chemotherapy. The data were randomly divided into two subsets with a 75:25 ratio using SPSS version 18.0 (SPSS, Chicago, IL, USA); one subset was divided into a low LMR group and a high LMR group for nomogram development, and the other was used for validation testing. A total of 501 normal volunteers (NVs) were also enrolled in the study. The inclusion criterion for the NVs was performance of a physical examination at Fujian Medical University Union Hospital, Fuzhou, China between December 2008 and December 2013. The exclusion criteria were as follows: (1) gastric cancer or malignant disease of other organs, (2) presence of coexisting hematological malignancies or disorders, (3) autoimmune disorders, (4) evidence of a severe inflammatory condition, and (5) recent steroid therapy. The ethics committee of Fujian Union Hospital approved this retrospective study. Written consent was obtained from the patients, and their information was stored in the hospital database and used for research.

### Blood sample analysis

Preoperative measurements of complete blood counts, including monocytes and lymphocytes, were performed within 7 d prior to surgery. LMR was calculated as the absolute value of the blood lymphocytes divided by the absolute value of the blood monocytes.

### Definition of the cut-off values

The X-tile program (http://medicine.yale.edu/lab/rimm/research/software.aspx) was used to determine the optimal cut-off values of LMR for overall survival (OS) and recurrence-free survival (RFS). The LMR cut-off value for both OS and RFS was 3.15 with maximum χ^2^ log-rank values of 44.84 (P<0.05) and 35.36 (P<0.05) [9] (Figure 1). Therefore, patients were categorized into two groups in the training set: 477 patients with low blood LMR (≤3.15) and 1,333 patients with high blood LMR (>3.15). The World Health Organization (WHO) defines an “elderly” or older person as an individual aged 65 years or older; therefore, we choose 65 years as the cut-off value for age [10]. Among the 1,810 patients, tumor size ranged from 5 to 180 mm, with a median of 50 mm. Therefore, we selected the median value of 50 mm as the cut-off value.

### Postoperative follow-up

The patients were monitored after surgery by telephone calls, outpatient visits and letters. OS was calculated as the number of months from the date of surgery to the date of last contact, date of death from any cause, or date of the study’s end point. RFS was calculated as the number of months from the date of surgery to the date of identification of disease recurrence (either radiological or histological), the date of death or last contact, or the date of the study’s end point. The follow-up time was calculated as from the date of surgery to the date of last contact, date of death from any cause, or date of study end point. All of the patients were regularly followed for at least 2 years except for those who died within 2 years after surgery.

### Statistical analysis

All enumeration and measurement data were analyzed using SPSS version 18.0 (SPSS, Chicago, IL, USA) and R 3.1.2 software (Institute for Statistics and Mathematics, Vienna, Austria). X-tile 3.6.1 software 20 (Yale University, New Haven, CT, USA) was used to determine the optimal cut-off values for blood LMR [9]. Chi-square, Fisher’s exact or unpaired Student’s t tests were used to compare the differences between groups in blood LMR and the clinicopathologic factors and the relationship between blood LMR and recurrence as appropriate. Survival curves were estimated using Kaplan–Meier methodology and were compared using the log-rank test. The Cox regression model was used to identify the variables that influenced OS and RFS. Multivariate analysis was performed using those variables that showed significant univariate relationships with OS and RFS. The significant variables from the multivariate analysis were included in the model. P<0.05 was considered to indicate statistical significance.

## SUPPLEMENTARY MATERIALS FIGURES


