# Supplementary material for: Preoperative lymphocyte-to-monocyte ratio as a strong predictor of survival and recurrence for gastric cancer after radical-intent surgery
Source: Oncotarget. 2017 Apr 12;8(45):79234–47. doi: 10.18632/oncotarget.17058 (PMC5668035; doi:10.18632/oncotarget.17058)
Supplement: Supplementary file 1 [file oncotarget-08-79234-s001.pdf]

Preoperative lymphocyte-to-monocyte ratio as a strong predictor of survival and recurrence for gastric cancer after radical-intent surgery

SUPPLEMENTARY MATERIALS

SUPPLEMENTARY FIGURES

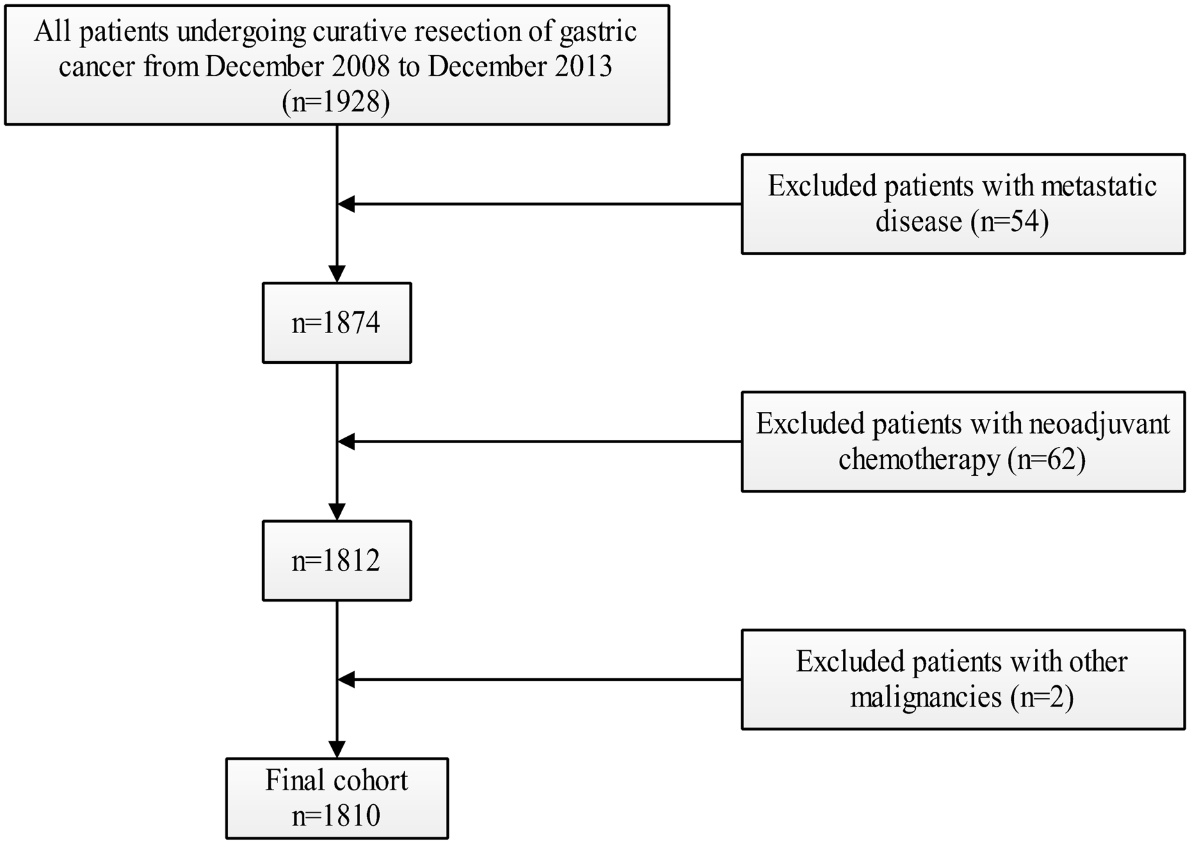

Supplementary Figure 1: Flow chart of the patient cohort based on inclusion and exclusion criteria.

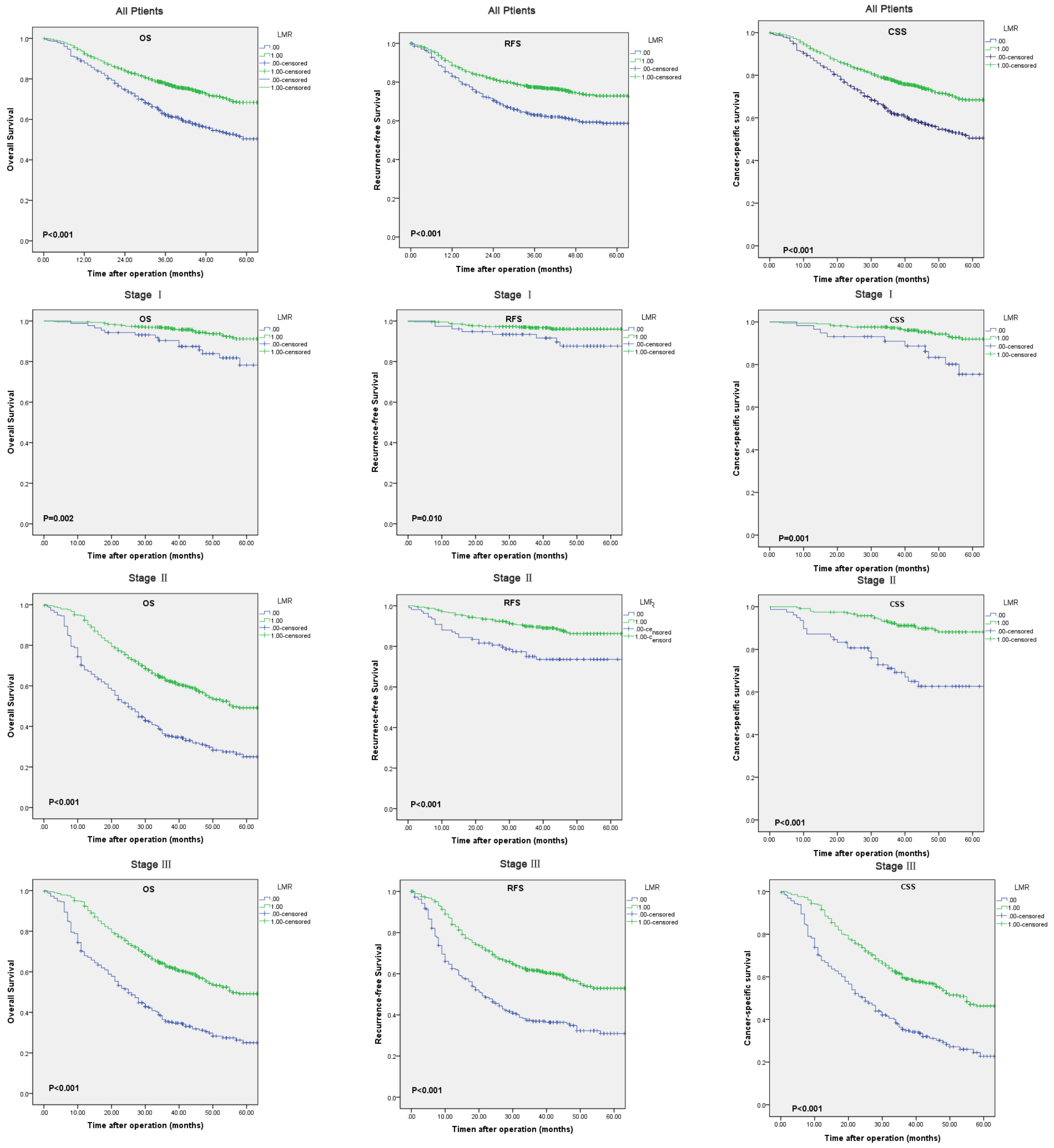

Supplementary Figure 2: A comparison of OS and RFS rates after gastrectomy between patient groups classified by LMR.

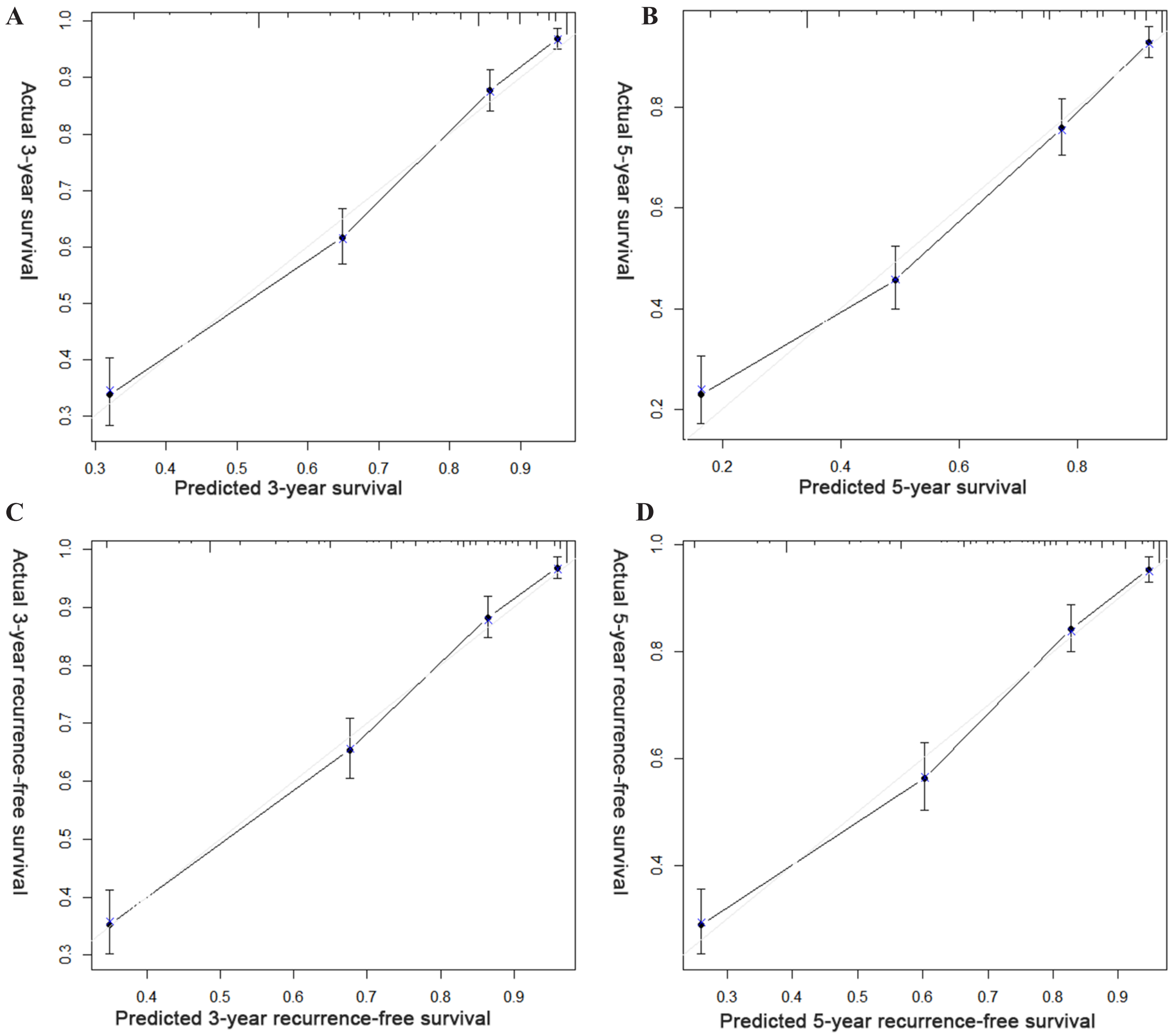

Supplementary Figure 3: Calibration curves for 3- and 5-year OS **(A** and **B)** and 3- and 5-year RFS **(C** and **D)** using nomograms revealed no deviations from the reference line**.**
